# Welcome to volume 10 of *Future Science OA*

**DOI:** 10.2144/fsoa-2023-0283

**Published:** 2024-06-12

**Authors:** Megan Bryant

**Affiliations:** 1 Future Science Group, Unitech House, 2 Albert Place, Finchley, London, UK, N3 1QB

## Introduction

To all our readers, Happy New Year and welcome to the first issue of the 10th volume of *Future Science OA*!

For every minute you spend reading this article, at least two new articles will enter the realm of biomedical literature [1]. In an era where information is abundant, ensuring that knowledge is accessible to all stands as a paramount goal. This commitment to accessibility has always been the hallmark of *Future Science OA.* As a fully gold open access journal, all our publications are freely available for anyone to read and are published with a CC BY licence, eliminating barriers that may have previously hindered the spread of knowledge.

In this foreword, we reflect on some of our notable content highlights from 2023 and provide an update for the exciting prospects you can expect from the journal in the year ahead.

## Content highlights

2023 has seen a dramatic increase in the number of submissions to *Future Science OA*. At the time of writing, we have already received a 300**%** increase in the number of submissions compared to 2022.

I am also delighted to share that this year marks a significant achievement for us as we have earned our first impact factor, which stands at a strong 2.5. In other rankings our 2022 CiteScore also saw an increase to 5.4. We would like to thank our authors and reviewers, as this achievement would not have been possible without their hard work and excellent research.

## Top content of 2023

Across ten issues, 75 papers were published in volume 9 of *Future Science OA*, encompassing a comprehensive range of cutting-edge topics. Our three most read articles were reviews on a diverse array of biomedical topics (Table 1). The review receiving the most downloads was Yoshinami and Shoji's ‘Recent advances in immunotherapy and molecular targeted therapy for gastric cancer’, which described the varying effectiveness of immune checkpoint inhibitors in different treatment settings, including first-line, second-line, perioperative and other experimental treatment approaches for advanced gastric cancer [2].

**Table 1.** Most read *Future Science OA* articles in 2023.

Rank	Title	Author(s)	Article type	Downloads	Ref.
1	Recent advances in immunotherapy and molecular targeted therapy for gastric cancer	Yuri Yoshinami and Hirokazu Shoji	Review	1170	[2]
2	Phytochemical and biological review of *Aegle marmelos* Linn	S Monika, M Thirumal and PR Kumar	Review	1105	[3]
3	Updates on drug designing approach through computational strategies: a review	Iqbal Azad, Tahmeena Khan, Naseem Ahmad, Abdul Rahman Khan and Yusuf Akhter	Review	841	[4]

Volume 9 featured 46 original research articles, each presenting pioneering discoveries within their specific fields. There are many examples of research articles published this year that demonstrated their impact. Our most highly read research article from Volume 9 of *Future Science OA* was Le Gall-David *et al.*‘s comparison of the efficacy of four DNA extraction kits for 16SrDNA microbiota profiling of diverse human samples [5].

Supplementing the research and review articles, *Future Science OA* also published eight added value content pieces, namely editorials and commentaries. The most highly read added value content included Abumsimir and Al-Qaisi's commentary on the next generation of malaria treatments, which amassed a total of 470 downloads [6].

**Figure 1.**
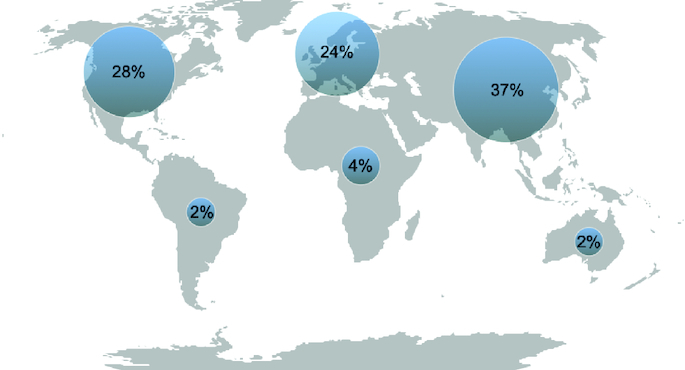
Topics covered in *Future Science OA* by percentage in 2023.

The range of topics published in *Future Science OA* has continued to grow throughout the year (Figure 1). This diversity reflects the dynamic nature of science and the ever-evolving landscape of biomedical research. To reflect this, this year we have increased our collections to include research on COVID-19 and dentistry.

## Maximizing discovery across the globe

**Figure 2.**
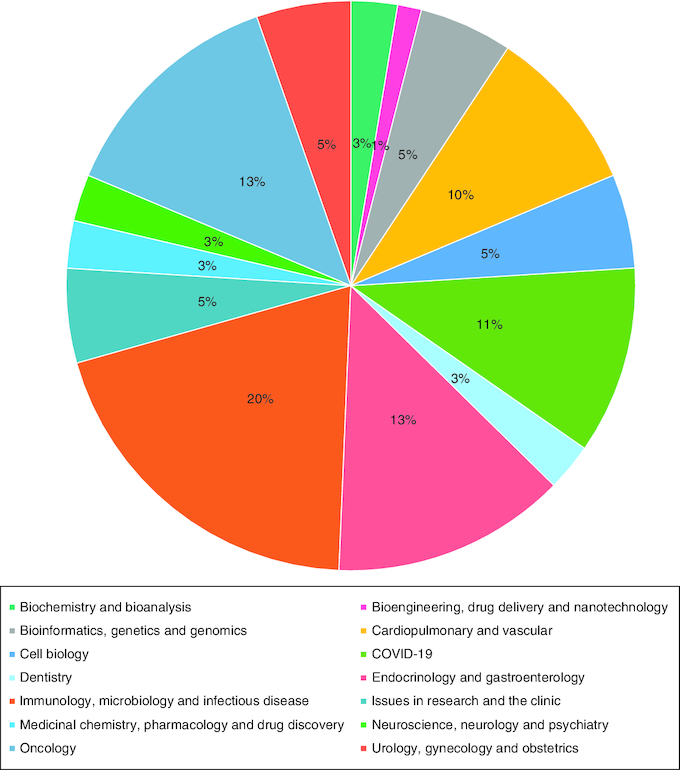
Percentage readership by location of access. Percentages are based on data collected from *Future Science OA* before 15 September 2023.

The diversity of our readers and authors mirrors the rich tapestry of the scientific field. This year, *Future Science OA* reached an extensive global audience, with readers spanning across more than 200 countries. Over the course of 2023, the journal accumulated a total of 181,562 online readers. Looking regionally, the largest volume of readers was based in North America, Europe and Asia (Figure 2). Nationally, the USA tops our readership, with a significant proportion of our 51,029 North American readers coming from the USA.

**Figure 3.**
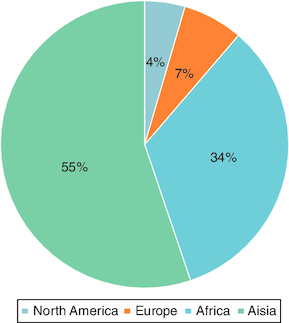
Demographic breakdown of authors by continent.

The geographical source of our published content is represented in Figure 3. This year, authors from a 25 countries made contributions to *Future Science OA*. We are encouraged to see an increase in the contribution of content from low- and middle-income countries. In fact, Tunisia had the highest contribution rate with 16 articles, closely followed by Jordan with ten and India with eight. Asia is our largest authorship demographic, constituting 55% of our published papers.

In line with our mission for accessibility and open science, we are delighted to continue offering full fee waivers for open access fees for authors that are geographically based in either Group A or Group B of the Research4Life list. We look forward to expanding our international authorship further throughout 2024, championing research from developing nations.

## Our growing editorial board

Our diverse editorial board bring a wealth of expertise to our team. In 2023, our editorial board saw the appointment of new editors, including Nihat Dilsiz and Luca Fiorillo.

Nihat currently is the Head of the Department of Molecular Biology and Genetics at the University of Health Sciences, Istanbul. His research involves exosomal microRNAs as biomarkers for early detection and effective treatment and drug development in various cancers and is particularly interested in the potential role of miRNAs in non-small cell lung cancer.

Luca specializes in rehabilitative dentistry, oral surgery and prosthodontics. In an exciting development facilitated by Luca's collaboration, we have recently had approval for a special focus issue on the topic of biomaterials within surgical dentistry. This forthcoming issue aims to provide a deep exploration of the biocompatibility and durability of various materials for dental surgical applications, including materials such as bioactive glass, nanomaterials and a combination of materials with stem cells. If you would like to contribute to this issue, please contact us.

Our editorial team would like to take this opportunity to thank all members of the editorial board for their enthusiasm and contributions in 2023. We look forward to working with you more in 2024.

## Looking forward to 2024

In the 10th volume, we hope that you will find a wide array of topics, perspectives and results that reflect the global nature of scientific research. We also look forward to our upcoming special focus issue, which will be the fourth in the journal's history.

So, here's to another year of research and making waves in the world of biomedical science. Thanks you for being part of this fantastic journey with us!

*Future Science OA* can be found across social media, and we are always excited to see our readers engage with us online. You can follow us on Twitter @fsgfso for highlights of the journal as well as all things biomedical science. As always, if you would like to get in touch about a potential article submission, offer feedback on the journal or anything else, just drop us an email or reach out to us on social media.
